# Randomized controlled trial of a positive emotion regulation intervention to reduce stress in family caregivers of individuals with Alzheimer’s disease: protocol and design for the LEAF 2.0 study

**DOI:** 10.1186/s12877-024-04817-5

**Published:** 2024-03-27

**Authors:** Caroline A. Leong, Amanda Summers, Veronika Grote, Kathryn Jackson, Glenna Dowling, Kari Snowberg, Paul Cotten, Elaine Cheung, DerShung Yang, Elizabeth L. Addington, Judith T. Moskowitz

**Affiliations:** 1 Department of Medical Social Sciences, Northwestern University, Chicago, Illinois USA USA; 2 Unversity of California, San Francisco, CA USA; 3 BrightOutcome, Chicago, IL USA; 4 Hinge Labs, New York, USA

**Keywords:** Positive affect, Dementia caregiving, Coping, Stress, Alzheimer’s disease, Randomized controlled trial

## Abstract

**Background:**

Caring for a loved one with Alzheimer’s disease can be stressful, resulting in poorer emotional and physical health among family caregivers. Although supportive resources for caregivers are available, distance, caregiver health, and the daily demands of caregiving are barriers to access. Based on research demonstrating the importance of positive emotions in coping with stress, our previous trial showed that dementia caregivers who participated in facilitated, web-based delivery of a positive emotion regulation intervention called LEAF (Life Enhancing Activities for Family caregivers) experienced increased positive emotion and decreased depression and anxiety. Building on this evidence, the LEAF 2.0 study aims to test whether web-based, self-guided delivery can confer similar benefits for caregivers of individuals with Alzheimer’s disease.

**Methods:**

This paper presents the design and methods for LEAF 2.0, a 3-arm web-based randomized controlled trial (*N* = 500) in which family caregivers of patients with Alzheimer’s disease (AD) are randomized to (1) the LEAF intervention facilitated remotely via the web (*N* = 200), (2) the LEAF intervention self-guided online (*N* = 200), or (3) an emotion reporting control (*N* = 100), which then crosses over to the intervention after approximately 6 months, half to the facilitated arm and half to the self-guided arm. We aim to (1) compare the effect of the facilitated and self-guided LEAF positive emotion interventions to an emotion reporting control condition on AD caregiver well-being (positive emotion, depression, anxiety, and perceived stress) and secondary outcomes (caregiving burden, caregiving self-efficacy, positive aspects of caregiving, quality of care, and AD patient quality of life); (2) assess whether effects are mediated by improvements in positive emotion or other aspects of caregiver well-being; and (3) test whether caregiver age or gender or the care recipient’s dementia severity moderates the effects of the intervention.

**Discussion:**

If demonstrated to be effective, LEAF can be widely disseminated and ultimately have a significant impact on the stress experienced by AD caregivers and the well-being of people living with Alzheimer’s disease.

**Trial Registration:**

ClinicalTrials.gov NCT03610698.

## Background

Alzheimer’s disease (AD) is a debilitating and anxiety-provoking illness that impacts not only those with dementia but also their loved ones, who often serve as caregivers. The prevalence of AD and other dementias is rising and is predicted to impact as many as 16 million Americans by 2050 [1]. More than 90% of persons with dementia (PWD) will receive some amount of care from a family member or friend [2]. Providing care for a PWD can be a chronic stressor and burden as the PWD loses the ability to perform many daily tasks themselves and comes to depend on the caregiver(s) to meet their needs. Furthermore, the psychological toll of seeing a loved one decline is substantial [3] and puts caregivers at a higher risk than noncaregivers for developing a variety of health issues [4–6], including higher levels of depression [7–9].

Existing interventions for dementia caregivers have mainly focused on reducing negative emotion and burden through teaching caregiving skills [10–12], providing social support [8], and teaching stress management techniques [13, 14]. However, a number of studies now show that increases in positive emotion are related to beneficial outcomes, independent of decreases in negative emotion [10, 11, 15–17], across a range of stressful contexts. Positive emotion and related constructs, such as optimism and resilience, have been linked to better mental health and quality of life and lower perceived burden in dementia caregivers [18, 19]. These findings suggest that a positive emotion regulation intervention could successfully decrease the impact of caregiving stress on the overall well-being of dementia caregivers and potentially increase the quality of care they provide for their loved one living with AD.

Prompted by the link between positive emotion and beneficial psychological and physical health outcomes, we developed LEAF (Life Enhancing Activities for Family Caregivers), an intervention that teaches evidence-based positive emotion regulation skills to caregivers to better cope with stress. It is premised on the Positive Pathways to Health model [20], which integrates evidence for positive psychological interventions (PPIs) and foundational theories, including Revised Stress and Coping Theory [7] and the Broaden-and-Build Theory of positive emotion [21]. The Positive Pathways to Health model posits that PPIs such as LEAF support well-being and stress management by increasing positive emotion, which provides a break from stressful experiences and builds physical, social, and intellectual personal resources that can renew one’s ability to cope. These proximal effects reduce stress, leading to improvements in health behaviors and physiologic functions, ultimately resulting in better psychological and physical health.

In the original LEAF study [22], dementia caregivers learned positive emotion regulation skills via one-on-one video sessions with a trained facilitator. Compared to an emotion reporting waitlist control condition, caregivers who were in the LEAF condition reported improvements in positive emotion, positive aspects of caregiving, depression, and anxiety. Furthermore, consistent with the Positive Pathways to Health model, positive emotion appeared to mediate the effect of the LEAF program on reduced depression [22].

Although LEAF 1.0 showed evidence of efficacy, the high-effort nature of one-on-one facilitated sessions limits our ability to disseminate the intervention on a larger scale. Thus, in the present trial (LEAF 2.0), we compare the effects of a self-guided online version of LEAF to the facilitated version. Primary outcomes are caregiver psychological well-being (positive emotion, depression, anxiety, perceived stress), and secondary outcomes are caregiving burden, caregiving self-efficacy, positive aspects of caregiving, quality of care, and AD patient quality of life. In addition, we explore whether the intervention’s effects on caregiver well-being are mediated through improvements in positive emotion. Finally, we examine whether caregiver age or gender or PWD dementia severity moderates the effects of the intervention on caregiver well-being.

## Methods/Design

### Overview of study design

LEAF 2.0 is a 3-arm, web-based, randomized controlled trial (*N* = 500) in which family caregivers of people with Alzheimer’s disease (AD) are randomly assigned to (1) the LEAF intervention facilitated remotely via the web (*N* = 200), (2) the LEAF intervention self-guided online (*N* = 200), or (3) an emotion reporting control (*N* = 100) that crosses over to the intervention after approximately 7 months, half to the facilitated arm and half to the self-guided arm.

### Participants

#### Eligibility criteria

To be eligible for participation in LEAF 2.0, participants must identify as the primary family caregiver of someone with Alzheimer’s disease or Alzheimer’s-related dementia and whose care recipient is not living in a care facility at the time of enrollment. “Primary family caregiver” is defined as the person who spends the most time caring for the individual with AD in a nonprofessional capacity. Participants must also be able to speak and read English, live in the United States, be at least 18 years of age, and have access to a reliable Wi-Fi connection. Respondents are ineligible if they have already participated in a prior version of LEAF.

#### Recruitment and enrollment

Participants are recruited online via advertisements on platforms such as Facebook and Google; postings in caregiver newsletters; and registries such as the Family Caregiver Alliance, Alzheimer’s Association TrialMatch, ResearchMatch, The New Normal (TNN), the Mesulam Center for Cognitive Neurology and Alzheimer’s Disease Clinical Core and its participants at Northwestern University. Additionally, we collaborate with Recruitment Partners LLC, a recruitment firm that specializes in recruitment for Alzheimer’s and related dementias research, to supplement our own efforts in meeting our recruitment goals. Advertisements contain a link to a short, online eligibility screener administered in REDCap. Individuals who are not eligible are immediately notified of their ineligibility and thanked for their time.

Eligible participants are contacted by study staff via email to sign up for Zoom audio calls for staff to confirm participant eligibility, explain the study and general research participation, and answer any questions the participant may have. Following the call, caregivers who wish to participate are sent an email with a link to an online consent form in REDCap. Upon completion of the consent form, REDCap automatically sends the participant a link to their baseline assessment.

### Procedures

#### Ethics approval and consent to participate

All procedures were approved and monitored by the Institutional Review Boards (IRB) at Northwestern University, and all participants provided informed consent electronically via REDCap. All staff members have completed Human Subjects Research Training either through the Collaborative Institutional Training Initiative or the NIH Human Subjects Training Module. The trial is registered at ClinicalTrials.gov (NCT03610698). Any protocol changes will be approved by IRB prior to implementing them, and information provided to participants and within the clinical trials registration will be updated accordingly.

#### Randomization

Participants are randomized into one of three arms using a 2:2:1 allocation ratio: (1) facilitated LEAF intervention condition with weekly one-on-one videoconferencing meetings via Zoom, (2) an online, self-guided LEAF intervention condition, or (3) an emotion reporting waitlist control that crosses over to either the facilitated or self-guided LEAF intervention after 7 months. We stratify randomization based on gender and level of caregiving burden using the short-form 6-item Zarit Burden Interview (ZBI) [23] as our measure of burden severity. The randomization assignments are computer-generated via a randomization schedule uploaded to REDCap.

All aspects of the study (recruitment, consent, intervention, and assessments) will be conducted online. Participants are given the option to receive an Android tablet, which enables the enrollment of caregivers who do not have access to a suitable device for participation. Those participants will be allowed to keep the tablet once they complete the study. Study staff preloaded the tablet with the Zoom videoconferencing app and a link to the study website on the home screen. Participants also receive tablet and Wi-Fi connection guides and additional tech support as needed from study staff. These measures increase the accessibility of the study for caregivers who describe themselves as less “tech-savvy.” No other participation incentives are provided.

#### Blinding/Concealment

There is no blinding/concealment of participants, research staff, or assessors in this study. It isn’t possible for participants to be blind to condition given the waitlist control design. Specifically, all participants receive the intervention, and there is no “placebo” or inert content. Arm 3, the emotion reporting waitlist control group, serves as the control condition for this study, and participants who are randomly assigned to that arm receive the intervention seven months after enrollment rather than right away. Assessments are self-administered via REDCap so there are no assessors to be blinded as to whether participants are in the intervention or control condition.

**Fig. 1:**
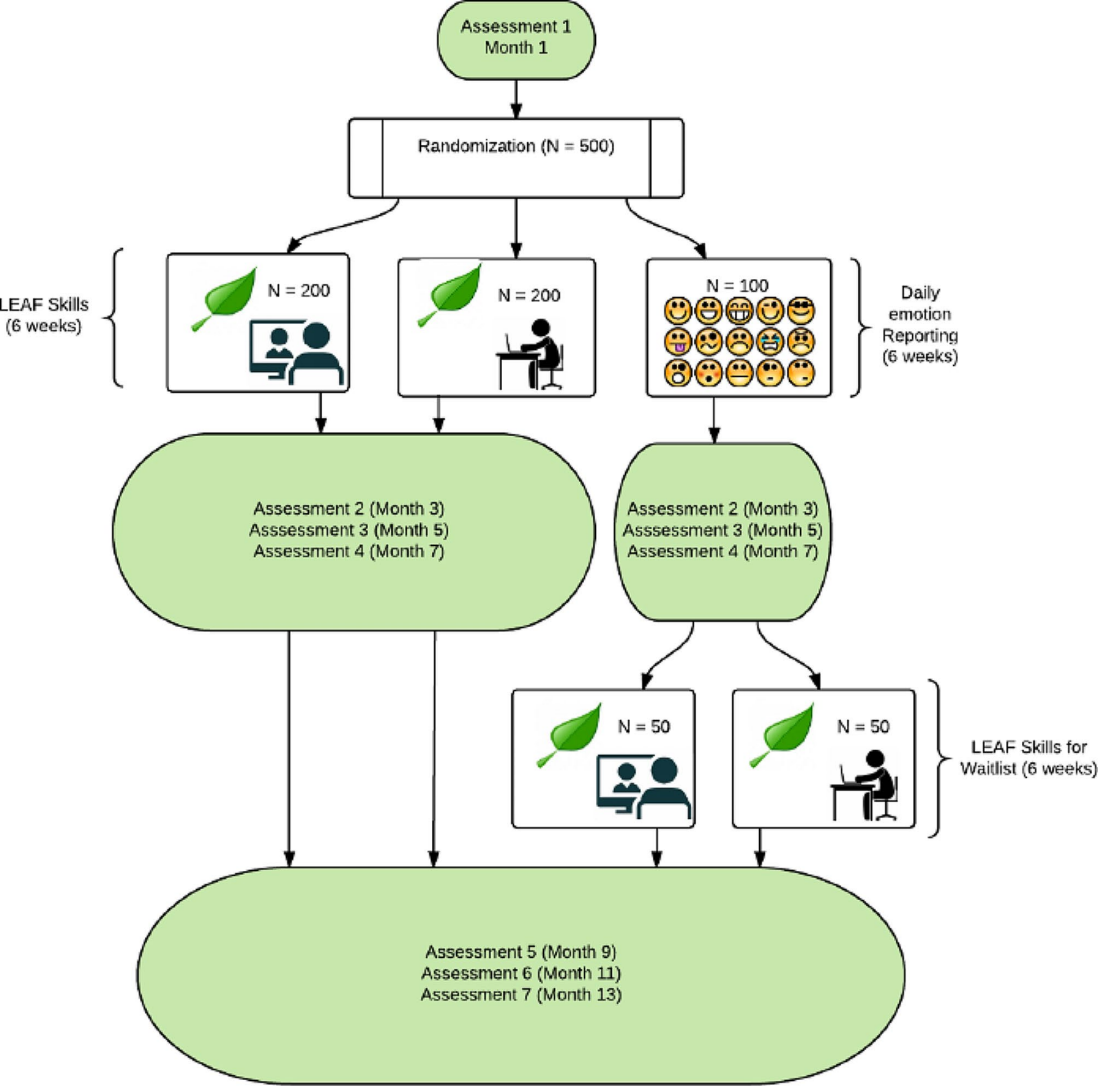
LEAF 2.0 participant timeline

#### Assessments

Online assessments are administered via REDCap, a secure, HIPAA-compliant, web-based app designed to support data collection and management for research studies. Participants complete assessments at seven time points (see Fig. 1): baseline and 3, 5, 7, 9, 11, and 13 months. Assessments include self-report measures of demographics, depression, positive and negative emotion, psychological well-being, coping resources, potential moderators, and satisfaction with the intervention. See Table 1 for the full list of measures. Participants also complete daily check-ins using surveys that include questions about stressful and positive events and positive and negative emotions. They complete these daily check-ins for a one-week “burst” each time they are sent an assessment and daily during the eight-week intervention period.

**Table 1:** Table of measures

Instrument	Description
Demographic and Clinical Characteristics	
Demographics	Collects information regarding caregiver age, length of time as a caregiver, relationship to care recipient, sex, race/ethnicity, marital status, education, and contact information of the primary care provider.
**Positive and Negative Emotions and Events**	
PROMIS Short Form v1.0 Positive Affect 15a [55]	Assess momentary positive or rewarding affective experiences, such as feelings and mood associated with pleasure, joy, elation, contentment, pride, affection, happiness, engagement, and excitement, over the past 7 days, using a scale of not at all to very much.
Modified Differential Emotions Scale: “Daily Emotion Check-in” (DES) [56]	Used to assess positive and negative emotion experienced within the last 24 h, modified to include additional positive affect and positive affectivity items, scored to create total positive and total negative affect scores.
Daily Inventory of Stressful Events (DISE) and Positive Events [57]	An end-of-day measure consisting of a brief set of stem and conditional questions in which participants report whether any of a series of stressful or positive events have occurred within the past 24 h.
**Psychological Well-Being**	
PROMIS SF v1.0-Depression 8a [58, 59]	Assesses depressive mood by having participants rate items focused on depressive symptoms over the past 7 days, using scale from Never to Always.
PROMIS SF v1.0- Anxiety 8a [58, 60]	Assesses various anxiety dimensions including fear, dread, hyperarousal, and somatic symptoms by having participants rate anxiety symptoms over the past 7 days using scale from Never to Always.
Profile of Emotional Competence Scale (PEC) [61, 62]	Self-reported measure of EC, identification, understanding, expression, regulation, and use of one’s own emotions and those of others.
Emotional Complexity Scale (ECS) [63]	Four item survey measuring the breadth of people’s emotions and their ability to differentiate between them.
Perceived Stress Scale (PSS-4) [64]	Used to measure how overloaded, unpredictable, and uncontrollable respondents perceive their lives to be. Scores range from 0 to 40, and higher scores indicate a higher stress level.
Satisfaction with Life Scale (SWLS) [65–67]	A short 5-item instrument designed to measure global cognitive judgments of satisfaction with one’s life.
PROMIS SF v1.0– Meaning and Purpose 8a [68, 69]	Assesses the degree to which one feels life has purpose and there are good reasons for living, including hopefulness, optimism, goal-directedness, and feelings that one’s life is worthy.
PROMIS SF v1.0– Sleep Disturbance 8b [70, 71]	Measures self-reported perceptions of sleep quality, depth, and restoration within the past 7 days, including difficulty falling asleep and staying asleep and sleep satisfaction.
Self-Compassion Scale (SCS)– Short Form [72]	Twelve-item measure used to assess a global self-compassion score.
Five Facet Mindfulness Questionnaire (FFMQ-15) [73]	Self-report scale to measure mindfulness with regards to thoughts, experiences, and actions in daily life.
**Caregiving Resources**	
Caregiving Status	Question that asks if participant is still current caregiver. Status may have changed if care recipient has moved into a care facility, the care recipient has passed away, or someone else has taken over as primary caregiver.
Zarit Burden Interview (ZBI-6) [23, 74, 75]	Assesses perceived burden in caregivers by assessing subjective feelings of the impact of caregiving on emotional and physical health, financial strain, and social functioning. Higher scores reflect greater burden.
Oberst Caregiving Burden (OCBS-15) [76]	Measures life changes as a consequence of caregiving with 15-items scored on a 7-point scale with responses ranging from “changed for the worse” to “changed for the best”.
Positive Aspects of Caregiving (PAC) [77]	Eleven item scale that identifies positive consequences of caregiving such as feeling more useful, feeling appreciated, and strengthening relationships with others. Higher scores indicate greater identification of the positives of being a caregiver.
Caregiving Mastery Subscale (CM)– Short Form [78]	Six-item measure used to assess a caregiver’s feelings about their own caregiving abilities.
Satisfaction with one’s own performance as a caregiver (SCQ) [79]	Twenty-seven item scale based on a family-crisis model that measures caregivers’ feelings of being capable of caring for an individual with dementia.
Dementia Severity Rating Scale (DSRS) [80]	Characterizes the level of functional abilities of the PWD being cared for by the study subject. The DSRS collects information from the caregiver on impairment severity of the PWD in 12 functional and cognitive ability domains. The full range of dementia severity is assessed, from no impairment observed to extreme impairment observed in each category.
Quality of Life of Care Recipient (QOL-AD) [81]	Thirteen items that provide caregiver report of the quality of life of persons who have been diagnosed with AD.
Revised Memory and Behavior Checklist (RMBC) [82]	A 24-item caregiver report measure of observable problems in the loved one with dementia.
**Moderators**	
PROMIS SF v1.1– Global Health [83]	Assesses participants’ perceptions of their health, quality of life, and abilities to carry out physical activities. The Global Health Scale contains 10 items that are rated on a scale of Excellent to Poor.
COMBO Health Behavior Measures [84]	Measures frequency of heath behaviors such as smoking, drinking, and physical activity.
Healthcare Utilization (HU)	Assesses degree to which individual is taking advantage of available health care resources.
Comorbidity checklist	Assesses other health conditions that may be co-occurring along with care recipient’s Alzheimer’s disease.
Medication Adherence, single item from Heart and Soul Study [85]	One question to assess if the individual is taking prescribed medications.
**Acceptability**	
Recommending the LEAF study	Assesses whether caregivers would recommend LEAF to another caregiver and/or friend.
Frequency of skill practice	Measures how often caregivers have been using each LEAF skill over the past month.
Modified Perceived Partner Responsiveness (mPPR) [86]	Three-item survey measures the degree to which people feel the study is responsive to them.
System Usability Scale (SUS) [87]	A ten-item questionnaire for measuring usability of the intervention, with participants marking statements about usability on a scale ranging from Strongly agree to Strongly disagree.
Device use and tech issues	Assesses which devices caregivers used most frequently for participation as well as sources and frequencies of any tech issues encountered.
Android tablet usage	Assesses for which aspects of the study the caregiver used the provided Android tablet, as well as frequency of usage.

#### Intervention and control conditions

##### Positive emotion regulation skills

This study utilizes a six-session, multicomponent positive emotion regulation skills intervention that includes eight empirically supported cognitive and behavioral skills that have been demonstrated to increase positive emotion across a number of samples [24–26]. The skills included in the LEAF 2.0 intervention are (1) noticing positive events, (2) savoring positive events, (3) gratitude, (4) mindful awareness and nonjudgment, (5) positive reappraisal, (6) self-compassion, (7) personal strengths, and (8) attainable goals (see Table 2). Giving participants a variety of skills to choose from allows them to identify the ones that are most effective for them, which may increase the chances that the intervention will be successful [27].

**Table 2:** Overview of intervention sessions, goals, and home practice

Skills	Background and Rational for Inclusion	Goals of session	Home Practice
Session 1: Positive Events, Savoring, Gratitude	Noticing positive life events is associated with increases in positive emotion and well-being [88–92], and may have a restorative effect on psychological resources used to combat and cope with stress [93]. Savoring a positive event, also known as capitalizing or amplifying, can prolong the positive emotional effect and work to strengthen the association between that event and the resulting positive emotion [93, 94]. Gratitude is a feeling of thankfulness and appreciation expressed toward something (e.g., person, spiritual entity, place). Studies in a variety of populations where participants kept a gratitude journal have found benefits including less negative emotion, increased positive emotion, better sleep quality, and greater satisfaction with life [95–97].	Identify positive events and the associated positive emotion; practice ways to savor the experience of positive events; and learn to practice gratitude.	Noting a positive event each day and writing about it (savoring); starting a daily gratitude journal and the daily emotion check-ins. The gratitude home practice continues through the rest of the intervention period.
Session 2: Everyday Mindfulness and Mindfulness Meditation	Mindfulness is defined as intentionally paying attention to one’s experience in the present moment without judgment [98]. Interventions aimed at increasing mindfulness are associated with increases in positive emotion [99].	Learn and practice the awareness and nonjudgment components of mindfulness.	Daily informal mindfulness activities, a 10-minute formal breath awareness activity, continuing the gratitude journal and daily emotion check-ins.
Session 3: Positive Reappraisal	Positive reappraisal is defined as reinterpreting an event’s significance in a more positive light. Stress and coping theory states that an individual’s appraisal of an event affects the degree to which that event is experienced as stressful [100]. Positive reappraisal is consistently associated with an increase in positive affect [101–104].	Understand positive reappraisal and the idea that different forms of positive reappraisal can all lead to increased positive emotion in the face of stress.	Reporting a relatively minor stressor each day, then listing ways in which the event can be positively reappraised. The daily formal mindfulness practice, gratitude journal, and the daily emotion check-ins.
Session 4: Self-Compassion	Self-compassion is extending warmth and understanding to oneself in the face of failure or suffering [30]. It has been shown to be associated with increased psychological well-being and positive affect [105]. We added self-compassion content for LEAF 2.0 based on our experience with previous caregiver studies [26] that indicated there was a need for participants to be less critical of themselves, and we anticipate that self-compassion will make it easier for participants to engage with the other skills in the program.	Learn about how to show compassion for oneself, especially in the context of caregiving. Understand how self-compassion relates to the other positive emotion skills in the LEAF 2.0 course.	Listing an act of self-compassion each day. The 10-minute mindful breathing, gratitude journal, and daily emotion check-ins continue.
Session 5: Personal Strengths, Attainable Goals	Identifying and focusing on one’s personal strengths is a type of self-affirmation that can lead to increased positive affect. Focusing on personal strengths is associated with better physical and psychological outcomes [106, 107]. *Attainable Goals*: Setting small, short-term goals is correlated with subjective reports of well-being, and perceptions of progress toward goals are associated with increased positive emotion as well as greater life satisfaction [108–110].	List personal strengths and note how they may have used these strengths recently; understand characteristics of attainable goals and set goals for the week.	Listing a strength each day and how it was “expressed” behaviorally, working toward one of the attainable goals and noting progress each day. The 10-minute mindful breathing, the gratitude journal, and the daily emotion check-ins continue.
Session 6: Skills Summary		Participants will receive a summary of the LEAF 2.0 course as a whole. Participants will identify which skills they enjoyed or did not enjoy and why. They will make a plan to practice the positive emotion skills beyond the LEAF 2.0 course.	Course wrap-up. The 10-minute mindful breathing, the gratitude journal, and the daily emotion check-ins continue.

LEAF 2.0 was tailored to include language and skills that would be most relevant to AD caregivers. The included skills are the same as LEAF 1 [26] and other previous versions of the intervention [25, 28, 29] with the exception of self-compassion, which was substituted for acts of kindness. Given that caregiving activities could be considered acts of kindness, we felt that self-compassion, defined as extending kindness to oneself (instead of self-judgment), especially during times of stress [30], was a better fit for these caregivers and made that change with this iteration of the LEAF program.

*Facilitator guided LEAF skills condition (Arm 1).* When a participant is randomized to Arm 1, they are assigned a facilitator who then schedules an initial videoconferencing session. The sessions take place over approximately 6 weeks (5 weeks for delivery of the skills plus one week for wrap-up and discussion of continued practice). The home practice follows each session for a total of 6 weeks. Each session has a didactic portion that covers 1–3 skills, followed by an opportunity to practice with the facilitator in the session. Between training sessions, participants are asked to complete home exercises and write daily experiences on the same online platform that the self-guided participants use (see below). They are also asked to complete daily stress and emotion questions included in the control condition.

LEAF sessions are facilitated by research staff and trainees who are not licensed clinicians. Facilitator training includes review of study aims and LEAF session guides (i.e., scripts), watching recordings of LEAF sessions conducted by seasoned facilitators, and role-play of LEAF sessions with an established facilitator acting as an AD caregiver. Training and feedback focus on presentation style, responding to typical challenges, and emphasizing consistent presentation of specific content milestones to maintain intervention fidelity. After new facilitators receive their first participant assignment, an established facilitator reviews their session recordings and provides feedback according to the quality assurance (QA) form and procedures (see below). Trained facilitators meet approximately monthly to review progress, share examples, and discuss challenges.

Following procedures used in our prior research [31], 10% of LEAF sessions are randomly selected for fidelity monitoring and QA. Video recordings are reviewed alongside the corresponding LEAF session script and a checklist of necessary session components. Reviews can also include qualitative comments about specific session components or the session overall. Each element on the QA checklist is assigned 1 point if present or 0 if missing, and the session score is calculated as a percentage. Any facilitator who averages less than 90% undergoes retraining.

*Self-guided condition (Arm 2).* In Arm 2, the skills are delivered as a self-guided program arranged into six online modules on the study platform. The web-based platform is designed, developed, and hosted by BrightOutcome, a digital health technology company that specializes in patient-centered healthcare applications. The website hosts the intervention for the self-guided arm, home practice for the facilitated and self-guided arms, and emotion reporting for the waitlist control condition.

**Fig. 2:**
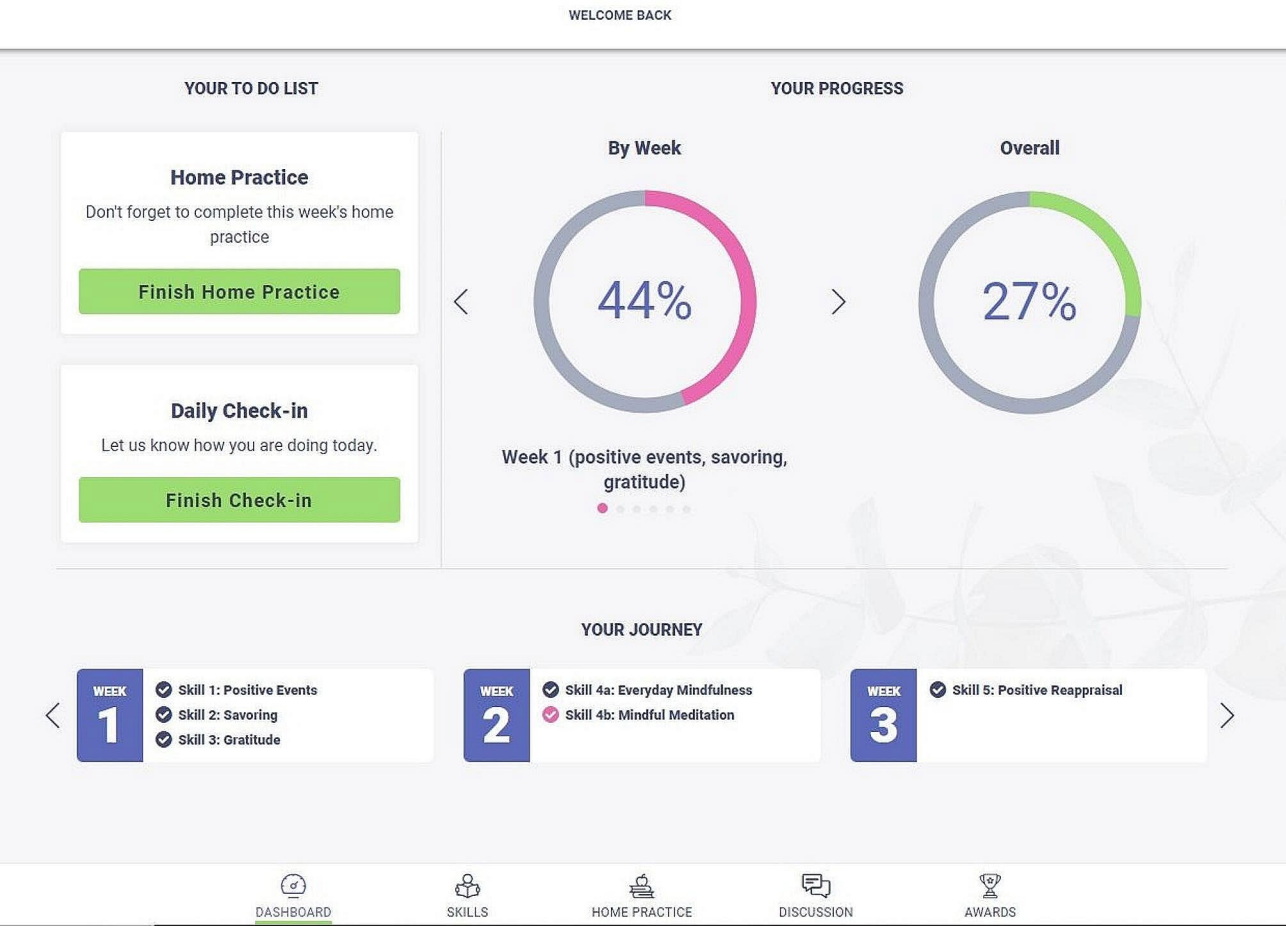
LEAF 2.0 website dashboard

**Fig. 3:**
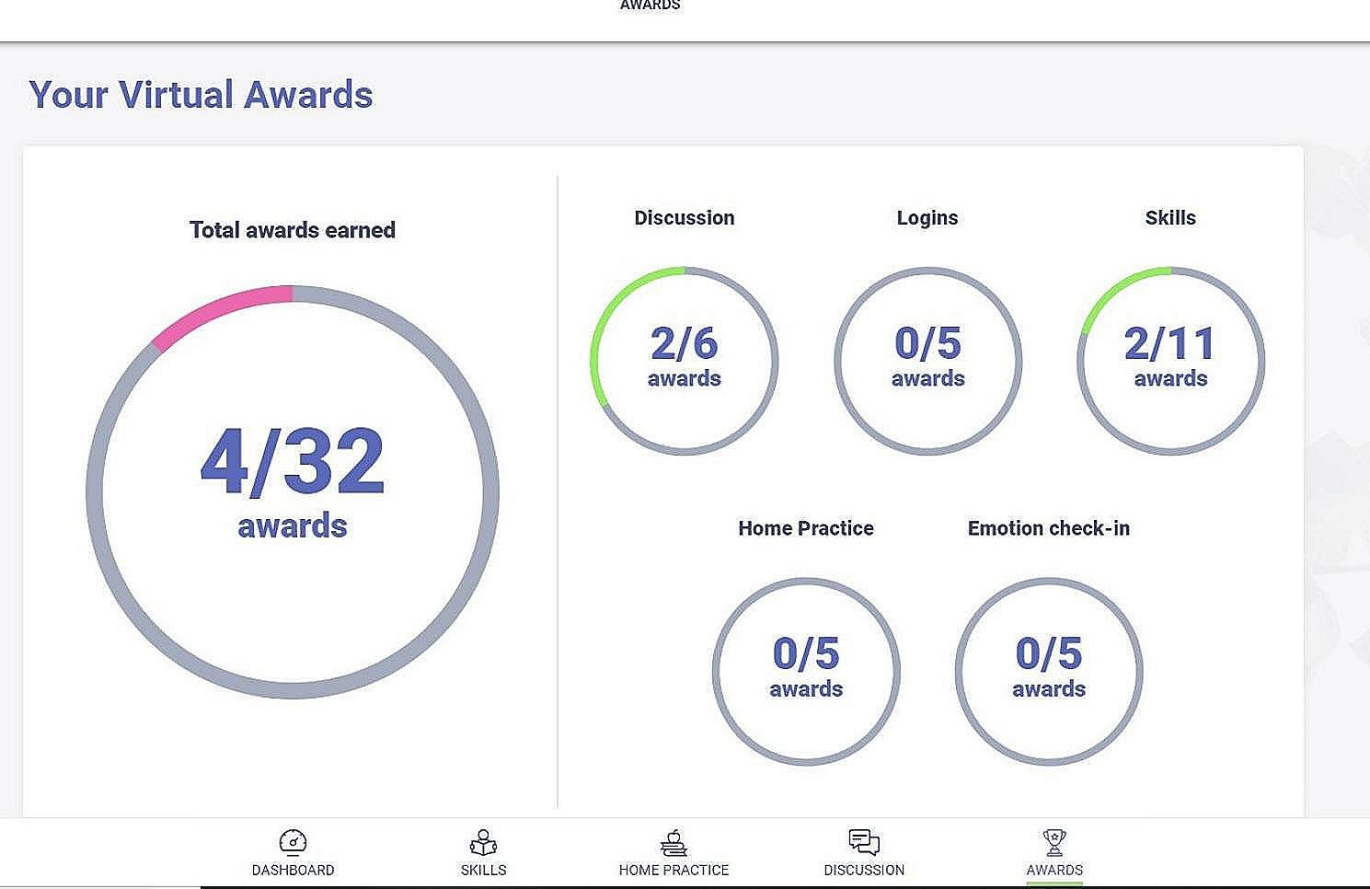
LEAF 2.0 website awards page

**Fig. 4:**
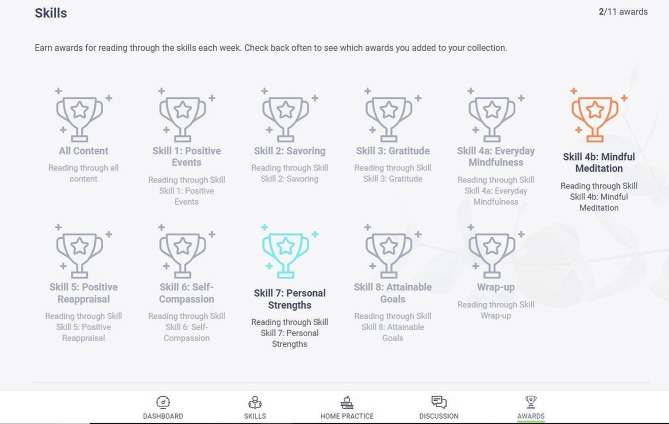
Example of LEAF 2.0 website virtual badges

Once randomized to the self-guided condition, participants are sent an email to activate their account on the LEAF study website, which includes features designed to increase both usability and participant engagement. The homepage of the LEAF website includes a dashboard with links to daily emotion check-in surveys and home practice activities, and a display of completion progress. For participants in the self-guided arm, the site includes a tab to easily navigate to the skill readings and a discussion board, where caregivers have the option of posting their home practice to share with fellow participants and reply to the posts of others. Self-guided participants earn virtual badges for various milestones throughout the intervention, such as logging in for ten consecutive days, posting to the discussion board five times, and completing ten home practices (see Figs. 2, 3 and 4).

Each lesson contains 1–3 skills and is designed to be completed within 1 week; however, to allow for variations in individual schedules and self-pacing, participants are given a total of 8 weeks to complete the LEAF course. Participants also have the opportunity to complete daily online home practice throughout the intervention, which includes daily emotion check-ins and skill practice exercises. The website remains available to participants even after their participation in the study has concluded, so they can continue the home practice or revisit skills.

*Waitlist Control (Arm 3).* Participants who are randomized into Arm 3 receive an automated email from REDCap notifying them that they will start the LEAF course in 7 months. The LEAF 2.0 platform sends an automated email instructing participants to activate their account, and they begin eight weeks of emotion reporting. Arm 3 participants begin their crossover to the intervention after completing the fourth assessment. Crossover procedures mirror the intervention group procedures described earlier, and the participant receives an email instructing them to reactivate their LEAF account.

##### Retention and attrition

Study staff provide participants with a contact email and phone number and respond promptly to any questions or concerns raised by participants. If participants fail to log into LEAF 2.0 to begin the intervention or emotion reporting, they receive up to three follow-up emails and/or phone calls to prompt engagement. Even if these contacts are ineffective, follow-up assessments are still sent unless the participant requests to withdraw.

Requests to withdraw from LEAF are processed by study staff, who note in REDCap the date the request was received, the reason (if given), the last activity the participant completed (e.g., completed session 2 with facilitator, completed assessment 4, etc.), and the name of the study team member who processed the withdrawal. The study team notifies the participant via email that their withdrawal has been completed and that they will not receive additional invites or reminders for study activities. Participants are permitted to keep their study-provided tablet upon withdrawing.

##### Data and safety monitoring

The funding agency did not require a Data and Safety Monitoring Board for this project because it is not an NIH-defined Phase III clinical trial and was deemed by the IRB to be of minimal risk. Instead, an external study monitor reviews results of interim analyses to determine whether group-level changes in PROMIS depression and anxiety raise study stopping concerns, based on meaningful increases ( > = 5 points on PROMIS T-score metric) or in the proportion of participants with moderate to severe elevations (PROMIS T-score > = 60) in these symptoms.

At the individual level of safety monitoring, LEAF staff will be alert to possible signs of significant distress and/or suicidality such as statements via phone, email, discussion board, or during facilitated sessions. When a staff member observes or receives a potential signal of increased distress in a participant, whether in response to study procedures that involve reporting on or describing negative or stressful emotions and experiences, the demands of caring for a loved one with AD, and/or the stressors of daily life, they will make note of as many details of the statement and/or circumstances as possible and contact the LEAF safety team, consisting of the study PI, team licensed clinical psychologist, and project director. With guidance from the PI and clinical psychologist, project director (and/or facilitator if participant is currently receiving facilitated LEAF sessions), will provide distressed participants with information about how to seek help. Given that participants may live anywhere in the United States, it will not be possible to provide them with direct referrals for therapy or medical care. However, study staff will provide resources for identifying a local provider or other support services, based on national recommendations (e.g., Substance Abuse and Mental Health Services Administration (SAMHSA)) or via their existing healthcare team (e.g., primary care physician) for further evaluation and local recommendations. If the participant’s distress appears to be related to their engagement in LEAF 2.0, the project director will also file Reportable New Information (RNI) with the IRB within five business days from the date when study staff are notified or become aware of the participant’s distress.

In addition, to protect participant confidentiality, identifiable information will be collected using REDCap which uses industry-standard encryption to protect participants’ information while in transit from the moment data is entered to the moment it is stored on HIPAA compliant servers. Access to REDCap is granted only to key personnel.

Bright Outcome hosts the LEAF 2.0 platform and is protected by end-to-end encryption and password measures. Participants will be assigned and identified by a unique Study ID on the LEAF 2.0 platform that only key IRB approved personnel will have access to. No identifiable data will ever be stored locally on staff computers, and no data are ever stored locally on participants’ devices (tablet, home computer, or mobile device).

Facilitated session recordings will be securely stored on Northwestern servers, accessible only to IRB-approved study staff. After completion of primary data analysis, recordings will be deleted.

#### Outcomes

##### Planned analyses

The primary outcomes, psychological well-being (positive emotion, depression, anxiety, perceived stress), will be evaluated using intent-to-treat longitudinal growth models with varying times of observation. Given variability in the actual time that participants will complete each assessment, we will use time elapsed since the baseline assessment (in months) as our metric of time. We plan to model time (centered at baseline) at Level 1 and the randomization arm (facilitator-guided LEAF vs. self-guided LEAF vs. emotion-reporting waitlist control) at Level 2, dummy-coded with emotion-reporting control as the reference category. We will test for both linear and quadratic changes over time and present the results for the best-fitting longitudinal change pattern. The primary parameters of interest will be the differences in the magnitude of change of each of the active intervention conditions (facilitator-guided LEAF and self-guided LEAF) relative to the control condition over time. We hypothesize that both active intervention conditions will demonstrate improved psychological well-being relative to the control. Intervention effects on secondary outcomes (see above and Table 1) will also be assessed with longitudinal growth models.

Mediation effects will be examined using parallel growth models [32] with individual varying times of observation. For the mediational analyses, we will combine the two active intervention conditions (facilitator-guided LEAF and self-guided LEAF) to explore the effects of the intervention (regardless of delivery method) compared to the control condition. We plan to conduct multilevel moderated mediation analyses [33] using a multilevel structural equation modeling (MSEM) framework [34]. More specifically, mediation effects will be estimated by examining the indirect effect of the intervention on change in caregiving burden through the effect of change in the mediating variable(s) (e.g., positive emotion) on change in caregiving burden. We will test the significance of the specific indirect effect in the MSEM using the Monte Carlo method with 20,000 bootstraps [35, 36]. In addition, we will conduct exploratory mediational analyses to explore whether improvements in positive emotion mediate the intervention effects on secondary psychological well-being (perceived stress, life satisfaction, self-efficacy, and meaning and purpose) and caregiving outcomes (positive aspects of caregiving, caregiving self-efficacy, quality of care provided, and care recipient QOL). Once again, we will conduct multilevel moderated mediation analyses to explore these effects.

Finally, a third set of analyses will utilize the postintervention assessments (Assessment 2 for Arms 1 and 2 or Assessment 5 for Arm 3) across both groups. Such analyses will enable us to examine whether benefits from the intervention are maintained over time. Therefore, the test of change for the two combined groups will be used to determine the shape of the trajectory for study participants following the end of the intervention and whether benefits to caregivers remain at the 1-, 3-, and 6-month follow-ups.

We will conduct exploratory tests to examine whether the effects of the intervention are moderated by age, gender, or baseline dementia severity. For the moderation analyses, we will combine the two active intervention conditions to explore the effects of intervention (regardless of delivery method) compared to the control condition. For each potential moderator, we will rerun the longitudinal growth models with a set of interaction variables between the moderator and the treatment indicator (LEAF intervention vs. emotion-reporting control) regressed on the slope factor (i.e., change over time). Due to power limitations, we will not test all four moderators in the same model or explore any interactions among the moderators.

##### Power

Simple group comparisons for single timepoints or pre-post comparisons between the facilitated (*n* = 200) and self-guided (*n* = 200) groups will have 80% power to detect effect sizes of *d* = 0.28 (R^2^ = 0.019), smaller than the observed effect sizes for positive emotion, negative emotion, stress, depression, and anxiety in our previous LEAF trial [37]. Comparisons between the wait-list control group (*n* = 100) and the LEAF interventions individually (*n* = 200) or taken together (*n* = 400) will have 80% power to detect effect sizes of *d* = 0.34 (R^2^ = 0.029) and *d* = 0.31 (R^2^ = 0.024), respectively. Our design of seven timepoints and 500 individuals exceeds established minimums for detecting predictors of individual differences [38] and for detecting associations between parallel growth processes [39]. Power for these models was estimated by Monte Carlo simulation to account for our longitudinal experimental design. The effects of the observed predictors (i.e., LEAF group, Aim 1) had 80% power for effects of *d* = 0.27 (R^2^ = 0.018). Parallel growth curves showed 80% power for slope correlations of *r* =.14 (R^2^ = 0.019) and comparable power for indirect effects of (*r* =.15, R^2^ = 0.021), consistent with previous work on mediational power [40].

##### Data access and dissemination

Prior to publication of results from the planned analyses (see above) only study staff will have access to the dataset. Manuscripts will be published in open access journals in accordance with guidelines for NIH-funded projects. The clinicaltrials.gov registration will also be updated with these findings. After publication of the planned analyses, the fully de-identified dataset will be made publicly available through the Open Science Framework (https://osf.io/).

## Discussion

The literature has repeatedly demonstrated the psychological and physical toll that caring for a loved one takes on an individual [41–48]. Given these adverse effects, there is a great need for interventions to improve family caregiver well-being; this need only grows as the prevalence of AD increases. LEAF aims to improve the lives of family caregivers by increasing positive emotion, which has been shown to have significant benefits not just for the caregiver but possibly for the PWD as well. LEAF 2.0 builds on our previous work [37] by increasing the availability and convenience of the LEAF program to a wider population of caregivers across the country. If the self-guided LEAF intervention is demonstrated to be as beneficial to caregivers as the facilitated version, LEAF 2.0 will prove to be an important step forward in both addressing the need for increased access to AD caregiver well-being resources and improving the quality of life of PWD and their family caregivers.

This study is not without limitations. While an online intervention can be convenient, it also limits the recruitable population of caregivers by excluding those who do not have reliable internet access or who are reluctant to participate because they are not comfortable with technology. We do attempt to address the latter through increased tech support throughout participation; however, low comfort with technology is still a formidable barrier [49, 50]. To the extent that access to a reliable internet connection or comfort with technology skews the demographics of caregivers who enroll in the study, our results will be similarly biased and will not be generalizable to the full array of AD caregivers.

In addition, the COVID-19 pandemic has been a significant obstacle in clinical trial recruitment (Mitchell et al., 2020). Recent studies make clear that COVID is having a negative impact on many aspects of the research enterprise across many types of studies, not simply those that relied on face-to-face recruitment [51–53]. Although LEAF 2.0 benefits from its virtual nature, the fact that caregivers face increased psychological distress and caregiving hours due to COVID-19 [54] may impact their inclination to participate in research and could further bias our sample to those who, perhaps, are less burdened in their caregiving.

Finally, the focus of LEAF on positive emotion should not be misconstrued as an attempt to minimize the significant stress of providing care for PWD. LEAF is not instructing caregivers to “don’t worry-be happy.” Instead, consistent with the Positive Pathways to Health Model [20], we argue that an intervention that specifically increases positive emotion can result in a cascade of beneficial effects, including reduced burden and improved quality of care. Ultimately, given the high levels of stress and depression documented in dementia caregivers, we consider positive emotion to be an inherently worthwhile goal.

## Data Availability

Not applicable.

## Abbreviations

AD: Alzheimer’s disease
LEAF: Life Enhancing Activities for Family Caregivers
MSEM: multilevel structural equation modeling
NIH: National Institutes of Health
PWD: persons with dementia
QOL: quality of life
RCT: randomized controlled trial
REDCap: Research Electronic Data Capture
ZBI: Zarit Burden Interview

## References

[1] Weuve J, Hebert LE, Scherr PA, Evans DA (2015). Prevalence of Alzheimer Disease in US States. Epidemiology.

[2] Kasper JD, Freedman VA, Spillman BC, Wolff JL (2015). The disproportionate impact of dementia on family and unpaid caregiving to older adults. Health Aff (Millwood).

[3] Perren S, Schmid R, Wettstein A (2006). Caregivers’ adaptation to change: the impact of increasing impairment of persons suffering from dementia on their caregivers’ subjective well-being. Aging Ment Health.

[4] Chakraborty R, Jana A, Vibhute VM (2023). Caregiving: a risk factor of poor health and depression among informal caregivers in India- A comparative analysis. BMC Public Health.

[5] Dowling GA, Merrilees J, Mastick J, Chang VY, Hubbard E, Moskowitz JT (2014). Life enhancing activities for family caregivers of people with frontotemporal dementia. Alzheimer Dis Assoc Disord.

[6] Schulz R, Eden J. National Academies of Sciences, Engineering, and Medicine (U.S.), editors. Families caring for an aging America. Washington, DC: The National Academies Press; 2016. 345 p. 27905704

[7] Folkman S (1997). Positive psychological states and coping with severe stress. Soc Sci Med.

[8] Mittelman MS, Haley WE, Clay OJ, Roth DL (2006). Improving caregiver well-being delays nursing home placement of patients with Alzheimer disease. Neurology.

[9] Sin NL, Lyubomirsky S (2009). Enhancing well-being and alleviating depressive symptoms with positive psychology interventions: a practice-friendly meta-analysis. J Clin Psychol.

[10] Chu P, Edwards J, Levin R, Thomson J (2000). The use of clinical case management for early stage Alzheimer’ patients and their families. Am J Alzheimers Dis Dementiasr.

[11] McCallion P, Toseland RW, Freeman K (1999). An evaluation of a family visit education program. J Am Geriatr Soc.

[12] Quayhagen MP, Quayhagen M (1989). Differential effects of family-based strategies on Alzheimer’s disease. Gerontologist.

[13] Morris RG, Woods RT, Davies KS, Berry J, Morris LW (1992). The use of a coping strategy focused support group for carers of dementia sufferers. Couns Psychol Q.

[14] Zarit SH, Anthony CR, Boutselis M (1987). Interventions with care givers of dementia patients: comparison of two approaches. Psychol Aging.

[15] Hébert R, Dubois MF, Wolfson C, Chambers L, Cohen C (2001). Factors associated with long-term institutionalization of older people with dementia: data from the Canadian study of Health and Aging. J Gerontol Biol Sci Med Sci.

[16] Mausbach BT, Chattillion E, Roepke SK, Ziegler MG, Milic M, von Känel R (2012). A longitudinal analysis of the relations among stress, depressive symptoms, leisure satisfaction, and endothelial function in caregivers. Health Psychol off J Div Health Psychol Am Psychol Assoc.

[17] Roepke SK, Allison M, Von Känel R, Mausbach BT, Chattillion EA, Harmell AL (2012). Relationship between chronic stress and carotid intima-media thickness (IMT) in elderly Alzheimer’s disease caregivers. Stress Amst Neth.

[18] Fianco A, Sartori RDG, Negri L, Lorini S, Valle G, Delle Fave A (2015). The relationship between burden and well-being among caregivers of Italian people diagnosed with severe neuromotor and cognitive disorders. Res Dev Disabil.

[19] Trapp SK, Perrin PB, Aggarwal R, Peralta SV, Stolfi ME, Morelli E (2015). Personal strengths and Health Related Quality of Life in Dementia Caregivers from Latin America. Behav Neurol.

[20] Moskowitz JT, Addington EL, Cheung EO (2019). Positive psychology and health: well-being interventions in the context of illness. Gen Hosp Psychiatry.

[21] Fredrickson BL (1998). What good are positive emotions?. Rev Gen Psychol.

[22] Moskowitz JT, Cheung EO, Snowberg KE, Verstaen A, Merrilees J, Salsman JM (2019). Randomized controlled trial of a facilitated online positive emotion regulation intervention for dementia caregivers. Health Psychol.

[23] Higginson IJ, Gao W, Jackson D, Murray J, Harding R (2010). Short-form Zarit Caregiver Burden interviews were valid in advanced conditions. J Clin Epidemiol.

[24] Addington EL, Cheung EO, Bassett SM, Kwok I, Schuette SA, Shiu E (2019). The MARIGOLD study: feasibility and enhancement of an online intervention to improve emotion regulation in people with elevated depressive symptoms. J Affect Disord.

[25] Moskowitz JT, Hult JR, Duncan LG, Cohn MA, Maurer S, Bussolari C (2012). A positive affect intervention for people experiencing health-related stress: development and non-randomized pilot test. J Health Psychol.

[26] Verstaen A, Moskowitz JT, Snowberg KE, Merrilees J, Dowling GA (2018). Life Enhancing activities for Family caregivers of people with dementia: protocol for a randomized controlled trial of a positive affect skills intervention. Open Access J Clin Trials.

[27] Schueller SM (2012). Personality fit and positive interventions: Extraverted and introverted individuals benefit from different happiness increasing strategies. Psychology.

[28] Moskowitz JT (2010). Positive affect at the onset of chronic illness: planting the seeds of resilience. Handbook of adult resilience.

[29] Cohn MA, Pietrucha ME, Saslow LR, Hult JR, Moskowitz JT (2014). An online positive affect skills intervention reduces depression in adults with type 2 diabetes. J Posit Psychol.

[30] Neff K (2003). Self-Compassion: an alternative conceptualization of a healthy attitude toward oneself. Self Identity.

[31] Moskowitz JT, Carrico AW, Duncan LG, Cohn MA, Cheung EO, Batchelder A (2017). Randomized controlled trial of a positive affect intervention for people newly diagnosed with HIV. J Consult Clin Psychol.

[32] Cheong J, Mackinnon DP, Khoo ST (2003). Investigation of mediational processes using parallel process latent growth curve modeling. Struct Equ Model Multidiscip J.

[33] Bauer DJ, Preacher KJ, Gil KM (2006). Conceptualizing and testing random indirect effects and moderated mediation in multilevel models: new procedures and recommendations. Psychol Methods.

[34] Preacher KJ, Zyphur MJ, Zhang Z (2010). A general multilevel SEM framework for assessing multilevel mediation. Psychol Methods.

[35] MacKinnon DP, Lockwood CM, Williams J (2004). Confidence limits for the Indirect Effect: distribution of the product and resampling methods. Multivar Behav Res.

[36] Preacher KJ, Selig JP (2012). Advantages of Monte Carlo Confidence Intervals for Indirect effects. Commun Methods Meas.

[37] Moskowitz JT, Cheung EO, Snowberg KE, Verstaen A, Merrilees J, Salsman JM (2019). Randomized controlled trial of a facilitated online positive emotion regulation intervention for dementia caregivers. Health Psychol off J Div Health Psychol Am Psychol Assoc.

[38] Rast P, Hofer SM (2014). Longitudinal design considerations to optimize power to detect variances and covariances among rates of change: Simulation results based on actual longitudinal studies. Psychol Methods.

[39] Hertzog C, Lindenberger U, Ghisletta P, von Oertzen T (2006). On the power of multivariate latent growth curve models to detect correlated change. Psychol Methods.

[40] Fritz MS, MacKinnon DP (2007). Required sample size to detect the mediated effect. Psychol Sci.

[41] Beach KD, Washburn EK, Gesel SA, Williams P (2021). Pivoting an Elementary Summer reading intervention to a virtual context in response to COVID-19: an examination of Program Transformation and outcomes. J Educ Stud Placed Risk JESPAR.

[42] Borelli WV, Augustin MC, de Oliveira PBF, Reggiani LC, Bandeira-de-Mello RG, Schumacher-Schuh AF (2021). Neuropsychiatric symptoms in patients with Dementia Associated with increased psychological distress in caregivers during the COVID-19 pandemic. J Alzheimers Dis JAD.

[43] Chattillion EA, Mausbach BT, Roepke SK, Von Känel R, Mills PJ, Dimsdale JE (2012). Leisure activities, caregiving demands and catecholamine levels in dementia caregivers. Psychol Health.

[44] Gouin JP, Glaser R, Malarkey WB, Beversdorf D, Kiecolt-Glaser J (2012). Chronic stress, daily stressors, and circulating inflammatory markers. Health Psychol off J Div Health Psychol Am Psychol Assoc.

[45] Kiecolt-Glaser JK, Marucha PT, Malarkey WB, Mercado AM, Glaser R (1995). Slowing of wound healing by psychological stress. Lancet Lond Engl.

[46] Schulz R, Beach SR (1999). Caregiving as a risk factor for mortality: the Caregiver Health effects Study. JAMA.

[47] Schulz R, Martire LM (2004). Family caregiving of persons with dementia: prevalence, health effects, and support strategies. Am J Geriatr Psychiatry off J Am Assoc Geriatr Psychiatry.

[48] Stall NM, Campbell A, Reddy M, Rochon PA (2019). Words Matter: the Language of Family Caregiving. J Am Geriatr Soc.

[49] Hayden LJ, Glynn SM, Hahn TJ, Randall F, Randolph E (2012). The use of internet technology for psychoeducation and support with dementia caregivers. Psychol Serv.

[50] Leng M, Zhao Y, Xiao H, Li C, Wang Z (2020). Internet-based supportive interventions for family caregivers of people with dementia: systematic review and Meta-analysis. J Med Internet Res.

[51] Maguire R, Hynes S, Seebacher B, Block VJ, Zackowski KM, Jonsdottir J (2021). Research interrupted: the impact of the COVID-19 pandemic on multiple sclerosis research in the field of rehabilitation and quality of life. Mult Scler J - Exp Transl Clin.

[52] Sathian B, Asim M, Banerjee I, Pizarro AB, Roy B, van Teijlingen ER (2020). Impact of COVID-19 on clinical trials and clinical research: a systematic review. Nepal J Epidemiol.

[53] Schnoll R, Bernstein SL, Kaufman A, Gross R, Catz SL, Cioe PA (2021). COVID-19 challenges confronted by Smoking Cessation clinical trials for people living with HIV: the experience of Grantees of the US National Cancer Institute. Nicotine Tob Res.

[54] Muldrew DHL, Fee A, Coates V (2022). Impact of the COVID-19 pandemic on family carers in the community: a scoping review. Health Soc Care Community.

[55] Salsman JM, Lai JS, Hendrie HC, Butt Z, Zill N, Pilkonis PA (2014). Assessing psychological well-being: self-report instruments for the NIH Toolbox. Qual Life Res Int J Qual Life Asp Treat Care Rehabil.

[56] Fredrickson BL. Chapter One - Positive Emotions Broaden and Build. In: Devine P, Plant A, editors. Advances in Experimental Social Psychology [Internet]. Academic Press; 2013 [cited 2024 Jan 23]. p. 1–53. Available from: https://www.sciencedirect.com/science/article/pii/B9780124072367000012.

[57] Almeida DM, Wethington E, Kessler RC (2002). The daily inventory of stressful events: an interview-based approach for measuring daily stressors. Assessment.

[58] Pilkonis PA, Choi SW, Reise SP, Stover AM, Riley WT, Cella D (2011). Item banks for measuring emotional distress from the patient-reported outcomes Measurement Information System (PROMIS®): depression, anxiety, and anger. Assessment.

[59] Pilkonis PA, Yu L, Dodds NE, Johnston KL, Maihoefer CC, Lawrence SM (2014). Validation of the depression item bank from the patient-reported outcomes Measurement Information System (PROMIS) in a three-month observational study. J Psychiatr Res.

[60] Schalet BD, Cook KF, Choi SW, Cella D (2014). Establishing a common metric for self-reported anxiety: linking the MASQ, PANAS, and GAD-7 to PROMIS anxiety. J Anxiety Disord.

[61] Brasseur S, Grégoire J, Bourdu R, Mikolajczak M (2013). The Profile of Emotional competence (PEC): development and validation of a self-reported measure that fits dimensions of emotional competence theory. PLoS ONE.

[62] Mikolajczak M, Brasseur S, Fantini-Hauwel C (2014). Measuring intrapersonal and interpersonal EQ: the Short Profile of Emotional competence (S-PEC). Personal Individ Differ.

[63] Wintre MG, Vallance DD (1994). A developmental sequence in the comprehension of emotions: intensity, multiple emotions, and valence. Dev Psychol.

[64] Jiang JM, Seng EK, Zimmerman ME, Sliwinski M, Kim M, Lipton RB (2017). Evaluation of the reliability, validity, and predictive validity of the subscales of the perceived stress scale in older adults. J Alzheimers Dis JAD.

[65] Diener E, Emmons RA, Larsen RJ, Griffin S (1985). The satisfaction with Life Scale. J Pers Assess.

[66] Emerson SD, Guhn M, Gadermann AM (2017). Measurement invariance of the satisfaction with Life Scale: reviewing three decades of research. Qual Life Res.

[67] Lorenzo-Seva U, Calderon C, Ferrando PJ, del Mar Muñoz M, Beato C, Ghanem I (2019). Psychometric properties and factorial analysis of invariance of the satisfaction with Life Scale (SWLS) in cancer patients. Qual Life Res.

[68] Salsman JM, Park CL, Hahn EA, Snyder MA, George LS, Steger MF (2018). Refining and supplementing candidate measures of psychological well-being for the NIH PROMIS®: qualitative results from a mixed cancer sample. Qual Life Res Int J Qual Life Asp Treat Care Rehabil.

[69] Salsman JM, Schalet BD, Park CL, George L, Steger MF, Hahn EA (2020). Assessing meaning & purpose in life: development and validation of an item bank and short forms for the NIH PROMIS®. Qual Life Res Int J Qual Life Asp Treat Care Rehabil.

[70] Buysse DJ, Yu L, Moul DE, Germain A, Stover A, Dodds NE (2010). Development and validation of patient-reported outcome measures for sleep disturbance and sleep-related impairments. Sleep.

[71] Yu L, Buysse DJ, Germain A, Moul DE, Stover A, Dodds NE (2011). Development of short forms from the PROMIS^™^ sleep disturbance and sleep-related impairment item banks. Behav Sleep Med.

[72] Raes F, Pommier E, Neff KD, Van Gucht D (2011). Construction and factorial validation of a short form of the Self-Compassion Scale. Clin Psychol Psychother.

[73] Christopher MS, Neuser NJ, Michael PG, Baitmangalkar A (2012). Exploring the Psychometric Properties of the five Facet Mindfulness Questionnaire. Mindfulness.

[74] Cheng ST, Kwok T, Lam LCW. Dimensionality of burden in Alzheimer caregivers: confirmatory factor analysis and correlates of the Zarit Burden interview. Int Psychogeriatr. 2014;1–9. 10.1017/S104161021400101X 24892872

[75] Schreiner AS, Morimoto T, Arai Y, Zarit S (2006). Assessing family caregiver’s mental health using a statistically derived cut-off score for the Zarit Burden interview. Aging Ment Health.

[76] Bakas T, Austin JK, Jessup SL, Williams LS, Oberst MT (2004). Time and difficulty of tasks provided by family caregivers of stroke survivors. J Neurosci Nurs J Am Assoc Neurosci Nurses.

[77] Tarlow BJ, Wisniewski SR, Belle SH, Rubert M, Ory MG, Gallagher-Thompson D (2004). Positive aspects of caregiving: contributions of the REACH Project to the development of New measures for Alzheimer’s caregiving. Res Aging.

[78] Struchen MA, Atchison TB, Roebuck TM, Caroselli JS, Sander AM (2002). A multidimensional measure of Caregiving Appraisal: validation of the Caregiver Appraisal Scale in Traumatic Brain Injury. J Head Trauma Rehabil.

[79] Jansen AP, van Hout HP, van Marwijk HW, Nijpels G, Gundy C, Vernooij-Dassen MJ (2007). Sense of competence questionnaire among informal caregivers of older adults with dementia symptoms: a psychometric evaluation. Clin Pract Epidemiol Ment Health CP EMH.

[80] Clark CM, Ewbank DC. Performance of the Dementia Severity Rating Scale: a Caregiver Questionnaire for Rating Severity in Alzheimer Disease. Alzheimer Dis Assoc Disord. 1996 Spring;10(1):31–9. 8919494

[81] Logsdon RG, Gibbons LE, McCurry SM, Teri L (1999). Quality of life in Alzheimer’s disease: patient and caregiver reports. J Ment Health Aging.

[82] Teri L, Truax P, Logsdon R, Uomoto J, Zarit S, Vitaliano PP (1992). Assessment of behavioral problems in dementia: the revised memory and behavior problems checklist. Psychol Aging.

[83] Hays RD, Bjorner JB, Revicki DA, Spritzer KL, Cella D (2009). Development of physical and mental health summary scores from the patient-reported outcomes measurement information system (PROMIS) global items. Qual Life Res.

[84] Fernald DH, Froshaug DB, Dickinson LM, Balasubramanian BA, Dodoo MS, Holtrop JS (2008). Common measures, Better outcomes (COMBO): a field test of brief Health Behavior measures in Primary Care. Am J Prev Med.

[85] Sin NL, Moskowitz JT, Whooley MA (2015). Positive Affect and Health Behaviors across 5 years in patients with Coronary Heart Disease: the Heart and Soul Study. Psychosom Med.

[86] Reis HT, Crasta D, Rogge RD, Maniaci MR, Carmichael CL. Perceived Partner Responsiveness Scale (PPRS). In: The Sourcebook of Listening Research [Internet]. John Wiley & Sons, Ltd; 2017 [cited 2024 Jan 23]. p. 516–21. Available from: https://onlinelibrary.wiley.com/doi/abs/10.1002/9781119102991.ch57.

[87] Brooke JSUS. A Quick and Dirty Usability Scale. In: Usability Evaluation In Industry. 1996.

[88] Folkman S, Moskowitz JT, Ozer EM, Park CL. Positive Meaningful Events and Coping in the Context of HIV/AIDS. In: Gottlieb BH, editor. Coping with Chronic Stress [Internet]. Boston, MA: Springer US; 1997 [cited 2024 Jan 23]. p. 293–314. (The Springer Series on Stress and Coping). 10.1007/978-1-4757-9862-3_11.

[89] Klaiber P, Wen JH, DeLongis A, Sin NL. The ups and downs of daily life during COVID-19: age differences in affect, stress, and positive events. J Gerontol B Psychol Sci Soc Sci. 2020;gbaa096. 10.1093/geronb/gbaa096 PMC7454856 32674138

[90] Lewinsohn PM, Amenson CS (1978). Some relations between pleasant and unpleasant mood-related events and depression. J Abnorm Psychol.

[91] Panaite V, Devendorf AR, Kashdan TB, Rottenberg J (2021). Daily life positive events predict well-being among depressed adults 10 years later. Clin Psychol Sci.

[92] Zautra AJ, Reich JW (1983). Life events and perceptions of life quality: developments in a two-factor approach. J Community Psychol.

[93] Langston CA (1994). Capitalizing on and coping with daily-life events: expressive responses to positive events. J Pers Soc Psychol.

[94] Bryant FB, Veroff J, Savoring US.; 2007 [cited 2024 Jan 23]. Available from: https://www.taylorfrancis.com/books/mono/10.4324/9781315088426/savoring-fred-bryant-joseph-veroff.

[95] Cregg DR, Cheavens JS (2021). Gratitude interventions: effective Self-help? A Meta-analysis of the impact on symptoms of depression and anxiety. J Happiness Stud.

[96] Emmons RA. Thanks! How the New Science of Gratitude can make you happier. Houghton Mifflin Harcourt; 2007. p. 268.

[97] Emmons RA, McCullough ME (2003). Counting blessings versus burdens: an experimental investigation of gratitude and subjective well-being in daily life. J Pers Soc Psychol.

[98] Kabat-Zinn J (2003). Mindfulness-based interventions in context: past, present, and future. Clin Psychol Sci Pract.

[99] Grossman P, Tiefenthaler-Gilmer U, Raysz A, Kesper U (2007). Mindfulness training as an intervention for fibromyalgia: evidence of postintervention and 3-year follow-up benefits in well-being. Psychother Psychosom.

[100] Folkman S, Stress. Appraisal and Coping. In: Gellman MD, Turner JR, editors. Encyclopedia of Behavioral Medicine [Internet]. New York, NY: Springer; 2013 [cited 2024 Jan 23]. p. 1913–5. 10.1007/978-1-4419-1005-9_215.

[101] Carver CS, Scheier MF (1994). Situational coping and coping dispositions in a stressful transaction. J Pers Soc Psychol.

[102] Rompilla DB, Hittner EF, Stephens JE, Mauss I, Haase CM (2022). Emotion regulation in the face of loss: how detachment, positive reappraisal, and acceptance shape experiences, physiology, and perceptions in late life. Emotion.

[103] Sears MR, Greene JM, Willan AR, Wiecek EM, Taylor DR, Flannery EM (2003). A longitudinal, population-based, cohort study of childhood asthma followed to adulthood. N Engl J Med.

[104] Wang K, Goldenberg A, Dorison CA, Miller JK, Uusberg A, Lerner JS (2021). A multi-country test of brief reappraisal interventions on emotions during the COVID-19 pandemic. Nat Hum Behav.

[105] McKay T, Walker BR (2021). Mindfulness, self-compassion and wellbeing. Personal Individ Differ.

[106] Quinlan D, Vella-Brodrick D, Gray A, Swain N. Teachers Matter: Student outcomes following a Strengths intervention are mediated by teacher strengths spotting. J Happiness Stud. 2019;20.

[107] Taylor SE, Lerner JS, Sherman DK, Sage RM, McDowell NK (2003). Are self-enhancing cognitions associated with healthy or unhealthy biological profiles?. J Pers Soc Psychol.

[108] Emmons RA (1986). Personal strivings: an approach to personality and subjective well-being. J Pers Soc Psychol.

[109] Emmons RA (1992). Abstract versus concrete goals: personal striving level, physical illness, and psychological well-being. J Pers Soc Psychol.

[110] Ouweneel E, Le Blanc PM, Schaufeli WB (2013). Do-it-yourself: an online positive psychology intervention to promote positive emotions, self-efficacy, and engagement at work. Career Dev Int.

